# A Critical Appraisal of the World Health Organization’s International Health Regulations (2005) in Times of Pandemic: It Is Time for Revision

**DOI:** 10.1017/err.2020.26

**Published:** 2020-04-03

**Authors:** Morten BROBERG

**Affiliations:** * University of Copenhagen, Faculty of Law, Copenhagen, Denmark; email: morten.broberg@jur.ku.dk.

## I. Epidemics are unavoidable – and dangerous

In 2010, when the world was hit by the so-called swine flu epidemic, Bill Gates, founder of Microsoft and philanthropist, wrote:

Hopefully this outbreak will serve as a wakeup call to get us to invest in better capabilities, because more epidemics will come in the decades ahead and there is no guarantee we will be lucky next time. The 1918 flu killed more than 50 million people.^1^

Every year, the US Intelligence Community provides a Worldwide Threat Assessment. Its January 2019 statement read:

We assess that the United States and the world will remain vulnerable to the next flu pandemic or large-scale outbreak of a contagious disease that could lead to massive rates of death and disability, severely affect the world economy, strain international resources, and increase calls on the United States for support.^2^

In September 2019, the independent Global Preparedness Monitoring Board observed that:

If it is true to say “what’s past is prologue”, then there is a very real threat of a rapidly moving, highly lethal pandemic of a respiratory pathogen killing 50 to 80 million people and wiping out nearly 5% of the world’s economy. A global pandemic on that scale would be catastrophic, creating widespread havoc, instability and insecurity. The world is not prepared.^3^

These are but three examples from the long list of warnings issued over the past few decades that the world should expect and prepare for the onset of devastating epidemics.

SARS-CoV-2 – the virus that causes the COVID-19 disease – supposedly emerged in the Hubei Province of China. The first human infection is thought to have been registered in November or December of 2019, pursuant to which the virus spread locally. On 31 December 2019, the UN World Health Organisation (WHO) was informed of “cases of pneumonia of unknown etiology (unknown cause)” detected in Wuhan City in the Hubei Province of China.^4^ In the first half of January 2020, cases of COVID-19 were registered in Thailand and Japan, signalling the beginning of the disease’s very rapid spread across the globe.

When it comes to coordination in the fight against contagious diseases, the WHO is the most important international actor. This article first presents the WHO’s legal basis for coordinating the work to counter epidemics (Section II). Subsequently, it identifies weaknesses in this legal basis (Section III) and points to solutions for remedying them (Section IV). The article ends with a few closing remarks (Section V).

## II. The World Health Organization’s tasks and competences in connection with epidemics

The WHO was established in 1948 and today counts practically all of the world’s countries as members. The explicit objective of the WHO is “the attainment by all peoples of the highest possible level of health”.^5^ To achieve this, the WHO is mandated to act as the directing and coordinating authority on international health work^6^ to furnish appropriate technical assistance and, in emergencies, necessary aid upon the request or acceptance of governments,^7^ and to stimulate and advance work to eradicate epidemic, endemic and other diseases.^8^ Within the WHO framework, the World Health Assembly (WHA) has been established as a decision-making body consisting of delegates from the WHO Member States.^9^ The WHA has the authority to adopt conventions and agreements with respect to any matter within the WHO’s competence.^10^ Moreover, the WHA has the authority to adopt regulations concerning sanitary and quarantine requirements and other procedures designed to prevent the international spread of disease.^11^

The WHO’s most important regulatory basis is the “International Health Regulations” – a set of rules whose origins can be traced back to the first International Sanitary Conference in 1851, at which several European states convened in an attempt to fight cholera and the first Sanitary Regulations were drafted. Following its establishment in 1948, the WHO used the International Sanitary Regulations. In 1969, the WHA amended and updated the rules and simultaneously changed their name to the International Health Regulations (IHR). IHR (1969) initially only covered six quarantinable diseases,^12^ which were subsequently reduced to just three: cholera, plague and yellow fever. Over time, it became clear that IHR (1969) suffered from serious shortcomings, in particular when it came to the fight against trans-border transmittable diseases. Firstly, too many transmittable diseases were not notifiable with the WHO. Secondly, IHR (1969) had been drafted so that the WHO was dependent on each state itself reporting the outbreak of a disease – and frequently the states simply abstained from doing this. Thirdly, there was an apparent lack of formalised and appropriate tools for the international coordination of the prevention of the spread of diseases. Shortly before the turn of the millennium, work towards a comprehensive review of IHR (1969) was initiated. The review gained considerable momentum when the deadly SARS epidemic broke out in 2003,^13^ and in 2005, the WHA adopted the presently applicable IHR (2005).^14^

IHR (2005) figures amongst the international agreements that most states have signed up to.^15^ Its purpose is “to prevent, protect against, control and provide a public health response to the international spread of disease in ways that are commensurate with and restricted to public health risks, and which avoid unnecessary interference with international traffic and trade”.^16^ For our purposes, it is of particular importance that the WHO’s members have undertaken to notify the WHO of events that may constitute what is called a “public health emergency of international concern”.^17^

When compared with IHR (1969), the adoption of IHR (2005) represents a crucial expansion in the rules’ coverage from three predefined transmittable diseases to any event that could be considered a “public health emergency of international concern” – and, as a matter of principle, this includes any outbreak of a transmittable disease. Just like IHR (1969), IHR (2005) relies primarily on notifications from WHO Member States. However, IHR (2005) also enables the WHO to examine possible “events” based on information received from sources other than the state where the event is supposed to have taken place; for example, information provided by the media, by researchers or by non-governmental organisations.^18^ Moreover, IHR (2005) enables the WHO to cooperate with other states and with other international organisations to fight disease outbreaks, even if the state where the outbreak originates is itself unwilling to cooperate.^19^

When the Chinese authorities realised that a virus epidemic had broken out, they implemented draconic measures to prevent the disease from spreading. In countries such as South Korea and Taiwan, it appears that the authorities have been able to contain the disease.^20^ By contrast, COVID-19 has spread dramatically in other countries, including Iran, Italy and Spain.

On 30 January 2020, the WHO’s Director General declared the COVID-19 outbreak a “public health emergency of international concern” (ie an extraordinary event determined to constitute a public health risk to other states through the international spread of disease and to which a coordinated international response could potentially be required).^21^ This allowed the WHO to issue so-called “temporary recommendations” such as specific health measures to be implemented by the state or states where the disease has broken out. It also allowed the WHO to issue “temporary recommendations” addressed to other states with regards to the exchange of persons and goods in order to prevent or reduce the international spread of the disease and to avoid unnecessary interference with international traffic.^22^ The temporary recommendations shall be recalled when they are no longer necessary – and, in any event, automatically expire three months after being issued.^23^ With regards to COVID-19, the WHO has issued several temporary recommendations, although the addressee states have not consistently complied with them.^24^

Experience from the WHO’s handling of previous epidemics clearly shows that IHR (2005) suffers from a number of weaknesses, and it seems only natural to expect that these weaknesses will also manifest themselves during the present COVID-19 pandemic.

## III. Weaknesses in the International Health Regulations (2005)

IHR (2005) entered into force on 15 June 2007. In the spring of 2009, the so-called swine flu epidemic began spreading throughout large parts of the world. For the first time, on 25 April 2009, the WHO’s Director General declared a “public health emergency of international concern”.^25^ However, this declaration elicited considerable criticism for giving rise to unnecessary fear, and several of the WHO’s members abstained from complying with its recommendations.^26^ To date, the WHO has declared a “public health emergency of international concern” on five occasions, the most recent of which was in connection to the spread of SARS-CoV-2.^27^

The situations deemed to constitute a “public health emergency of international concern” have demonstrated the shortcomings of the instruments available to the WHO. This was arguably most clearly illustrated in connection with the 2014 Ebola epidemic. Subsequent examinations of the handling of this epidemic are unanimously critical and go so far as to maintain that better preparedness and a faster, more coordinated response may have prevented most of the 11,000 deaths directly attributed to Ebola, as well as the broader economic, social and health crises that ensued from the epidemic.^28^

In the efficient prevention of a transboundary epidemic, it is crucial that WHO Member States comply with recommendations so as to ensure a coordinated response. However, examinations of the responses to the WHO’s handling of different epidemics show that, as a general rule, states only half-heartedly follow recommendations and compliance with IHR (2005).^29^ There are three principal reasons to explain this lack of mobilisation. Firstly, several Member States simply do not have the requisite resources to follow the rules. Secondly, certain states are either unable or unwilling to quickly notify the WHO of disease outbreaks. Thirdly, WHO Member States may introduce travel and trade restrictions of their own, even if these initiatives may be unnecessary or may conflict with the recommendations of the WHO.

With regards to the lack of resources, Article 5(1) of IHR (2005) establishes that Member States “shall develop, strengthen and maintain … the capacity to detect, assess, notify and report events” within the framework of the WHO, and it lays down deadlines for these requirements to be met. If a Member State is unable to fulfil the requirements, it may ask the WHO for assistance.^30^ However, several states have still been unable to comply with the requirements and, among them, some have not even been able or willing to carry out a satisfactory (mandatory) self-assessment of their abilities to fulfil IHR (2005) requirements.^31^ Against this background, several proposals have been put forward as to the best way of encouraging governments to allocate sufficient means to ensure the fulfilment of their surveillance obligations in relation to outbreaks of transmittable diseases. These proposals include offering technical assistance or development assistance for the implementation of programmes, exertion of diplomatic pressure or a requirement for the International Monetary Fund to take “outbreak preparedness” into account when preparing their “country reports” (given that a negative country report increases the cost of borrowing money for said country).

With regards to delays in notifying the WHO of disease outbreaks (as required under IHR (2005)), this can almost certainly be attributed to the fact that this information has the potential to harm both national tourism and trade.^32^ Thus, there is a need to identify ways of offsetting the adverse consequences of reporting outbreaks and thereby counterbalance many states’ innate reluctance towards rapid reporting.^33^

States’ reluctance towards rapid reporting of disease outbreaks is intimately connected to the third challenge; namely, that states not directly affected by the disease outbreak have shown themselves ready to introduce pre-emptive travel and trade restrictions against the reporting state, even insituations where the WHO has made it clear that such restrictions are not objectively justifiable.^34^ When a state experiences a disease outbreak and then becomes subject to such travel and trade restrictions, it will be faced with a negative impact on its financial situation. In particular for a financially weak state, it may therefore be entirely rational to refrain from reporting an outbreak – even though it is, in principle, an obligation to do so under IHR (2005) – and instead hope that it does not develop into an epidemic. IHR (2005) expressly requires that measures addressing disease outbreaks interfere as little as possible with world traffic, a stance reflected in the WHO’s recommendations. Nevertheless, examples abound of states and businesses introducing disproportionate restrictions vis-à-vis those states that have reported disease outbreaks. This inevitably makes it more difficult to convince states experiencing disease outbreaks to speed up the reporting process.

## IV. Remedying the weaknesses in the International Health Regulations (2005)

Although IHR (2005) constitutes a quantum leap forward in comparison to IHR (1969), it nevertheless suffers from significant weaknesses. It has been posited that IHR (2005) has been drafted on the basis of an assumption that contagious diseases break out in poor countries in the Global South so that the objective behind the international regulation of such diseases is to protect wealthy countries in the Global North from the spread of these diseases.^35^ This assumption is, however, misleading in two respects. Firstly, contagious diseases do not only break out in the Global South. However, because states in the Global South, on average, have fewer resources to enable them to detect and respond to transmittable diseases at an early stage, there is a higher risk that if such diseases do break out, they may quickly become unmanageable in these country contexts. Secondly, in the contemporary world, transboundary relations are so ubiquitous that if a transmittable disease runs out of control, it will be very difficult for any state to keep it outside of its borders. Indeed, the spread of COVID-19 is testament to this.

On the surface level, IHR (2005) sets out to detect and contain potentially transmittable diseases as early as possible in ways that affect the free exchange of people and goods as little as possible. But reality is much more complicated. Firstly, IHR (2005) presupposes that the affected states have sufficient resources to detect, assess, notify and report (possible) outbreaks of transmittable diseases. In reality, many states do not have these resources, and the longer it takes between the outbreak of a transmittable disease and the time at which it is “detected, assessed, notified and reported”, the more likely it is that it starts spreading uncontrollably. Secondly, there is a clear cost–benefit asymmetry between, on the one hand, the state or states where the outbreak originates and, on the other, virtually all other states. Thus, if an event (a disease) warranting notification arises in a given state, this state must allocate resources to first comply with the IHR (2005) surveillance requirements and thereupon to fight the disease outbreak. In addition, this state may expect adverse economic consequences due to a drop in foreign tourism and less international trade. In contrast, if the affected state manages to contain the disease internally, it will be to the benefit of all other states.

Arguably, the solution is to make it both possible and attractive for all states to efficiently combat any event that may develop into a transboundary epidemic, but this solution will be costly.^36^ Firstly, financial and human resources will need to be made available to those states that themselves lack the ability to comply with IHR (2005), and the WHO must be able to call on skilled healthcare professionals who can be deployed at very short notice, whenever and wherever the need arises. Secondly, we must ensure that there is a positive economic incentive for all states to comply with IHR (2005). Thus, rather than punishing states for not complying with IHR (2005) and thereby encouraging them to suppress information about notifiable diseases, we should reward the weakest states for complying with IHR (2005). Both of these measures are costly and imply the allocation of substantial amounts of additional funding to the WHO on a continuous basis.

## V. Closing remarks

In 1918, the world was hit by the Spanish flu – an influenza virus that is estimated to have infected about 500 million people, the equivalent of one quarter of the Earth’s total population at the time. Estimates of the number of people who died from this disease range from 17 million to 100 million. The Spanish flu came in three waves. The first wave arrived in the spring of 1918 and killed a very high number of people. In autumn of 1918, the second wave washed across the globe. Those who had survived the first wave had become immune, but the virus had mutated between the first and the second waves to become significantly more lethal. A third wave followed in the winter/spring of 2019.

According to the reports on COVID-19, the virus is extremely contagious, but only a small proportion of those infected fall seriously ill, and fewer still die. But the very high number of people infected means that even this small proportion of seriously ill patients places a very considerable strain upon our hospitals. It is easy to see that if the virus retains its ability to spread, but at the same time mutates so as to become much more lethal, we will be moving from chaos towards Armageddon.^37^ Hopefully, this will not happen. However, the COVID-19 pandemic provides a warning bell that is too loud to be ignored and reminds us that it is high time to prepare ourselves against those transmittable diseases that will hit us in the future; in particular, we must ensure that the WHO is much better equipped to lead this fight.

## Footnotes

1 B Gates, “A Better Response to the Next Pandemic”, blog post, 18 January 2010 <https://www.gatesnotes.com/Health/A-Better-Response-to-the-Next-Pandemic>.

2 “Statement for the Record – Worldwide Threat Assessment of the US Intelligence Community”, 29 January 2019, p 21 <https://www.dni.gov/files/ODNI/documents/2019-ATA-SFR---SSCI.pdf?fbclid=IwAR12sbZkI5cS9l9frjX4dnpCGe7bm1JkImi6rW-ykvX4qLBe8cuY5Mn8_8s>.

3 Global Preparedness Monitoring Board, “A World at Risk: Annual Report on Global Preparedness for Health Emergencies”, World Health Organization, Geneva 2019 <https://apps.who.int/gpmb/assets/annual_report/GPMB_annualreport_2019.pdf>.

4 Cf WHO, “Novel Coronavirus (2019-nCoV) Situation Report – 1”, 21 January 2020 <https://www.who.int/docs/default-source/coronaviruse/situation-reports/20200121-sitrep-1-2019-ncov.pdf?sfvrsn=20a99c10_4>.

5 WHO Constitution, Art 1.

6
ibid, Art 2, letter a.

7
ibid, Art 2, letter d.

8
ibid, Art 2, letter g.

9
ibid, Arts 9 and 10.

10
ibid, Art 19.

11
ibid, Art 21, letter a.

12 Cholera, plague, relapsing fever, smallpox, typhus and yellow fever.

13 Cf WHA resolution 56.29 of 28 May 2003 (Severe acute respiratory syndrome (SARS)).

14 With regards to the adoption of IHR (2005), see LO Gostin , Global Health Law (Cambridge, MA, Harvard University Press 2014 ) ch 6.

15
ibid, p 181.

16 IHR (2005), Art 2.

17 Cf IHR (2005) Art 6(1). “Public health emergency of international concern” has been defined in Annex 2 to IHR (2005).

18 IHR (2005), Arts 9 and 10.

19
ibid, Arts 10(4) and 14.

20 With particular regard to Taiwan’s handling of COVID-19, see CJ Wang, CY Ng and RH Brook, “Response to COVID-19 in Taiwan – Big Data Analytics, New Technology, and Proactive Testing” (2020) JAMA, 3 March 2020 <https://jamanetwork.com/journals/jama/fullarticle/2762689>.

21 IHR (2005), Art 1(1).

22
ibid, Art 15(2). Measures may address travel, trade, quarantine, screening and treatment.

23
ibid, Arts 15(1) and (3) and 49.

24 Cf J Zarocostas, “What Next for the Coronavirus Response?” (2020) 395 The Lancet 401.

25 Cf Swine influenza, Statement by WHO Director-General, Dr Margaret Chan, 25 April 2009 <https://www.who.int/mediacentre/news/statements/2009/h1n1_20090425/en>.

26

LO Gostin , MC DeBartolo and EA Friedman , “The International Health Regulations 10 Years On: The Governing Framework for Global Health Security” (2015) 386 The Lancet 2222 10.1016/S0140-6736(15)00948-4PMC713806426615327.

27 The five cases are: polio (2014), Ebola (2014), Zika (2016), Ebola (2019) and SARS-CoV-2 (2020).

28

S Moon et al, “Post-Ebola Reforms: Ample Analysis, Inadequate Action” (2017) 356 BMJ j280.2811531610.1136/bmj.j280

29
ibid.

30 Cf IHR (2005), Art 5(3).

31 Cf Gostin et al, supra, note 26, 2224; Moon et al, supra, note 28, 356–57.

32 See likewise B Bennett and T Carney , “Public Health Emergencies of International Concern: Global, Regional, and Local Responses to Risk” (2017) 223 Medical Law Review 234 10.1093/medlaw/fwx004PMC710766628379440. The two authors argue that in global health governance it is important to clearly define roles and allocate responsibilities and accountabilities, and they find that in order to ensure that global health governance of risk management for infectious diseases will not remain patchy and imperfect, it is important to ensure “clarity of responsibilities”, as well as “the associated accountability and enforcement mechanisms necessary to ensure that countries are meeting their obligations under the IHR”.

33 The World Bank has developed a so-called “Pandemic Emergency Financing Facility” that offers poor countries the possibility of receiving financial assistance if they are hit by an influenza or coronavirus pandemic. See further World Bank, “Fact Sheet: Pandemic Emergency Financing Facility”, 20 March 2020 <https://www.worldbank.org/en/topic/pandemics/brief/fact-sheet-pandemic-emergency-financing-facility>.

34 Gostin et al, supra, note 26, 2224–25.

35 Cf Gostin, supra, note 14, 179.

36 The question as to how to improve the WHO and IHR (2005)’s ability to prevent transboundary epidemics has been the topic of several studies that have provided almost identical answers. See, for example, Global Preparedness Monitoring Board, supra, note 3; and S Moon et al, “Will Ebola Change the Game? Ten Essential Reforms before the Next Pandemic. The Report of the Harvard–LSHTM Independent Panel on the Global Response to Ebola” (2015) 386 The Lancet 2204.10.1016/S0140-6736(15)00946-0PMC713717426615326

37 It has been estimated that about 2% of all persons who have been infected by SARS-CoV-2 in the period 2019–2020 have died. This can be compared with the fatality rate of the Ebola virus, which, since 1976, has been estimated to be about 50%; or with MERS between 2012 and 2019, where an estimated 34% of those infected died; or finally with the SARS outbreak in 2003, where an estimated 10% of infected patients died; cf E Mahase , “Coronavirus: Covid-19 Has Killed More People than SARS and MERS Combined, Despite Lower Case Fatality Rate” (2020) 368 BMJ m641 3207106310.1136/bmj.m641; WHO, “Fact-sheet, ‘Ebola virus disease’”, 10 February 2020 <https://www.who.int/news-room/fact-sheets/detail/ebola-virus-disease>.

